# Silicon Induces Heat and Salinity Tolerance in Wheat by Increasing Antioxidant Activities, Photosynthetic Activity, Nutrient Homeostasis, and Osmo-Protectant Synthesis

**DOI:** 10.3390/plants12142606

**Published:** 2023-07-10

**Authors:** Ansa Aouz, Imran Khan, Muhammad Bilal Chattha, Shahbaz Ahmad, Muqarrab Ali, Iftikhar Ali, Abid Ali, Fatmah M. Alqahtani, Mohamed Hashem, Tasahil S. Albishi, Sameer H. Qari, Muhammad Umer Chatta, Muhammad Umair Hassan

**Affiliations:** 1 Department of Agronomy, University of Agriculture Faisalabad, Faisalabad 38040, Pakistan; ansasheikh22@gmail.com (A.A.);; 2 Department of Agronomy, Faculty of Agriculture Sciences, University of the Punjab, Lahore 54000, Pakistan; 3 Department of Entomology, Faculty of Agriculture Sciences, University of the Punjab, Lahore 54000, Pakistan; 4 Department of Agronomy, Muhammad Nawaz Shareef University of Agriculture, Multan 66000, Pakistan; 5 School of Life Sciences & Center of Novel Biomaterials, The Chinese University of Hong Kong, Shatin, Hong Kong; 6 Department of Genetics and Development, Columbia University Irving Medical Center, New York, NY 10032, USA; 7 Department of Agricultural and Food Sciences, University of Bologna, 40127 Bologna, Italy; 8 Department of Biology, College of Science, King Khalid University, Abha 61413, Saudi Arabia; 9 Biology Department, College of Applied Sciences, Umm Al-Qura University, Makkah 21955, Saudi Arabia; 10 Department of Biology, Al-Jumum University College, Umm Al-Qura University, Makkah 21955, Saudi Arabia; 11 Research Center on Ecological Sciences, Jiangxi Agricultural University, Nanchang 330045, China

**Keywords:** antioxidants, chlorophyll, growth, nutrient homeostasis, reactive oxygen species, yield

## Abstract

Modern agriculture is facing the challenges of salinity and heat stresses, which pose a serious threat to crop productivity and global food security. Thus, it is necessary to develop the appropriate measures to minimize the impacts of these serious stresses on field crops. Silicon (Si) is the second most abundant element on earth and has been recognized as an important substance to mitigate the adverse effects of abiotic stresses. Thus, the present study determined the role of Si in mitigating adverse impacts of salinity stress (SS) and heat stress (HS) on wheat crop. This study examined response of different wheat genotypes, namely Akbar-2019, Subhani-2021, and Faisalabad-2008, under different treatments: control, SS (8 dSm^−1^), HS, SS + HS, control + Si, SS + Si, HS+ Si, and SS + HS+ Si. This study’s findings reveal that HS and SS caused a significant decrease in the growth and yield of wheat by increasing electrolyte leakage (EL), malondialdehyde (MDA), and hydrogen peroxide (H_2_O_2_) production; sodium (Na^+^) and chloride (Cl^−^) accumulation; and decreasing relative water content (RWC), chlorophyll and carotenoid content, total soluble proteins (TSP), and free amino acids (FAA), as well as nutrient uptake (potassium, K; calcium, Ca; and magnesium, Mg). However, Si application offsets the negative effects of both salinity and HS and improved the growth and yield of wheat by increasing chlorophyll and carotenoid contents, RWC, antioxidant activity, TSP, FAA accumulation, and nutrient uptake (Ca, K, and Mg); decreasing EL, electrolyte leakage, MDA, and H_2_O_2_; and restricting the uptake of Na^+^ and Cl^−^. Thus, the application of Si could be an important approach to improve wheat growth and yield under normal and combined saline and HS conditions by improving plant physiological functioning, antioxidant activities, nutrient homeostasis, and osmolyte accumulation.

## 1. Introduction

Salinity stress (SS) is a serious threat to crop production and global food security. The extent of soil salinity is continuously increasing, and every year, 4 × 10^4^ hectares around the globe lose their ability to produce crops, owing to SS [1,2]. Globally, 6% of lands are salt-affected, which accounts for 20% of total cultivated lands, and out of which 50% of lands are irrigated [3,4]. The excessive salts in the rhizosphere induce a deleterious impact on crop growth and development [5]. Soil salinity has adverse impacts on wheat crops and causes water shortage, reduces photosynthetic rates and nutrient uptake, and increases the uptake of toxic ions, which in turn decreases wheat growth and yield [6]. Further, SS also reduces the leaf area and root and shoot growth, and induces the excessive production of reactive oxygen species (ROS), which cause significant damage to the wheat crop [7,8]. Moreover, SS also disturbs the photosynthetic process in wheat by decreasing chlorophyll contents, denaturing enzymes, and damaging photosynthetic apparatus and creating negative water potential [9,10]. However, wheat plants have developed an excellent enzymatic defense system, and they also accumulate the potential osmolytes and hormones to mitigate the adverse impacts of SS [11].

The global climate is continuously warming with the corresponding increase in atmospheric temperature and carbon dioxide (CO_2_) concentration; as a result, plants are adversely affected by these changes [12]. Heat stress (HS) refers to an increased atmospheric temperature that affects plants’ physiological and biochemical processes. Wheat crops are very sensitive to HS and this stress negatively affects wheat growth and yield [13,14,15]. Heat stress also decreases chlorophyll synthesis and electron transport and damages the photosynthetic apparatus, which results in reduced assimilate production, thereby resulting in significant yield losses in wheat [16]. Moreover, HS also induced ROS production, which damages enzymes, proteins, and nucleic acid; induces stomata closing; and increases leaf temperature, which causes a significant reduction in final productivity [17]. Additionally, HS also causes substantial yield losses in wheat by impairing pollen vitality, pollen tubes, and seed settings and inducing flower abortion [18].

The research on combined stresses indicates that plants act differently and evoke distinct integrated signaling networks, which result in different responses, as compared to single stresses [19]. The results of combined stresses could be neutral, additive, or synergistic [20]. Nonetheless, in plants, common pathways and responses are also shared by stress combinations, and thus, diverse physiological and molecular reactions occur in plants. For instance, HS impairs plant enzymatic activities, which create a metabolic imbalance that affects plant growth as a result [21,22]. On the other hand, SS-induced Na^+^ toxicity reduces nutrient uptake, causes deleterious impacts on plant genes and enzymes, and causes various metabolic deficiencies [23]. Moreover, combined heat and saline stress can inhibit the root and shoot growth and RWC and can increase the MDA and EL, as well as Na+ to a significant extent [24,25].

Silicon (Si) is the second most common element found in the Earth’s crust (27.2%) after oxygen, and it is considered a beneficial element for plants [26]. Silicon is widely used to alleviate abiotic stress in wheat and the application of Si improves tolerance against abiotic stress by increasing water uptake, antioxidant activities, osmolyte accumulation, and nutrient uptake [27,28]. Si has been recognized to improve antioxidant activities, maintain ionic balance and the K⁺/Na⁺ ratio, and reduce ROS, which improves SS tolerance in wheat [29,30]. Si nutrition has been found to improve plant growth, biomass production, and the root and shoot growth of wheat by reducing Na^+^ and Cl^−^ uptake and partitioning Na^+^, Cl^−^, and mineral ions [31]. Furthermore, Si also improves K^+^ uptake, chlorophyll synthesis, osmolyte and hormone accumulation, and antioxidant activities, which improve wheat growth under saline conditions [32]. Further, Si also triggers signaling molecules and stress-associated genes and improves antioxidant activities and osmolyte accumulation, which in turn alleviate the adverse impact of HS on wheat [33]. Additionally, Si prevents the degradation of cellular proteins, improves osmolyte accumulation, prolongs the stay-green trait, improves photosynthetic activities, and reduces MDA and ROS accumulation, which ensures better wheat growth under HS [34].

Wheat is an important food crop and a staple food of many nations; however, it is considered to be sensitive to salinity and heat stress. Salinity and heat stresses can cause a significant reduction in the growth and yield of wheat [35,36]. Here, in this study, we evaluated the response of SS and HS, both alone and in combination on f diverse wheat genotypes under Si application. We hypothesized that wheat genotypes may vary in their ability to tolerate SS and HS. We also hypothesized that the application of Si would be able to alleviate the deleterious impacts of SS and HS by improving physiological functions, antioxidant activities, osmolyte accumulation, and nutrient homeostasis. Therefore, the present study aimed to determine the effect of Si on growth, physiological and antioxidant activities, nutrient homeostasis, and the grain yield of diverse wheat cultivars growing under SS and HS and a combination of both stresses. 

## 2. Material and Methods

### 2.1. Growth Conditions and Plant Materials

A pot experiment was conducted in the greenhouse of University of Agriculture Faisalabad to determine the effect of exogenously applied silicon in mitigating heat and salt stress in different wheat cultivars. The soil used for filling the pots (5 kg capacity) was collected from an agronomic field (31.8 °N, 73.8 °E, 184 m asl) previously sown with a rice crop. The soil was collected from the upper 0–20 cm layer and had a 7.88 pH, organic matter 1.22%, EC 0.99 d ms^−1^, total nitrogen (N) 0.63%, and available phosphorus (P) and potassium (K) at 6.91 and 158 ppm, respectively. The concentration of sodium chloride (NaCl) required for achieving the 8 dS m^−1^ EC value was calculated using the following formula.

$$\mathrm{NaCl}\;\mathrm{required}\;(\frac{g}{\mathrm{kg}})=\frac{TSS*58.5*Saturation\;\left(\%\right)}{100*1000}.\;$$

TSS indicates total soluble salts, and these were calculated by multiplying EC differences (required soil EC–initial soil EC) with 10. NaCl was added at a rate of 8.24 g/kg to obtain the required EC (8 dSm^−1^). To determine the soil saturation percentage (SSP), 250 g of soil was utilized, and soil paste was prepared by adding the distilled water. Soil paste was kept at room temperature for 2 h, and extract was obtained and SSP was determined via the following equation:

$$saturation\;\left(\%\right)\frac{loss\;in\;soil\;weight\;on\;drying}{weight\;of\;soil\;after\;drying}\times 100$$

The sodium silicate was used as a source of Si and was applied at a rate of 500 mg kg^−1^ of soil [37]. Si was applied at the time of the filling of pots and was thoroughly mixed with the soil. Furthermore, heat stress was applied to pots by placing them in a greenhouse at the reproductive stage for two weeks.

### 2.2. Experimental Treatments

This study encompasses different wheat genotypes, namely Akbar-2019, Subhani-2021, and Faisalabad-2008, as well as different treatments: control, salt stress (8 dSm^−1^), heat stress, salt stress + heat stress, control + Si, salt stress + Si, heat stress+ Si, and salt stress + heat stress + Si. The present study was carried out in a completely randomized design under a factorial arrangement (3 × 8 × 3) with three replications. The pots were examined regularly, and plants were watered based on visual observation. Moreover, fertilizers urea (1.46 g), di-ammonium phosphate (DAP) (1.56 g), and sulfate of potassium (1.10 g) were applied together to fulfill NPK requirements. P and K were applied as a basal dose by mixing fertilizers in the soil, while N was applied to two splits at the sowing and tillering stages.

### 2.3. Measurement of Growth Traits

Three plants were randomly chosen from each pot, and their heights were measured and averages were taken. Likewise, three plants were selected and their root and shoot lengths and dry weights were measured.

### 2.4. Determination of Photosynthetic Pigments, Leaf Relative Water Content, and Electrolyte Leakage

Chlorophyll and carotenoid contents were determined using the method of Arnon [38]. Fresh samples (0.5 g) of plants were homogenized in 80% methanol solution to obtain the extract, and then the extract was centrifuged and filtered, and absorbance was noted at 645, 480, and 663 nm wavelengths to determine the chlorophyll a and b and carotenoid contents. To determine relative water content (RWC), fresh leaf samples were taken from the plant and weighed to determine fresh weight (FW), then the sample leaves were submerged in water for 24 h, and after removal from water, the turgid weight (TW) was taken. Thereafter, turgid leaf samples were oven-dried (70 °C) until constant weight was reached in order to determine dry weight (DW), and RWC was determined via the following procedure: RWC (%) = FW − DR/TW − DR × 100. For the determination of electrolyte leakage (EL), 0.5 g of fresh leaf samples were submerged in water for 30 min, after which EC_1_ was measured, and then the samples were heated for 50 min and EC_2_ was measured. The final electrolyte (EL) values were determined through the following equation: EL = EC_1_/EC_2_ × 100.

### 2.5. Determination of Osmolytes and Oxidative Stress Markers

For the determination of total soluble protein (TSP), frozen leaf samples were ground in 5 mL phosphate buffer and then centrifuged at 14,000 rpm for 15 min at 4 °C. Then, the plant samples were treated with 2 mL Bradford reagent, and this mixture was allowed to sit for 15–20 min and absorbance was noted at 595 nm [39]. For total free amino acid (FAA) determination: 1 mL of crude extract was homogenized with a buffer, poured into test tubes, and 1 mL pyridine with 1 mL ninhydrin was added. Then, these test tubes were placed in a water bath for 30 min at 90 °C at a volume of 25 mL, and absorbance was noted at 570 nm to determine FAA [40]. In order to determine H_2_O_2_ concentration, 0.5 g of samples was ground with trichloroacetic acid (TCA: 0.5 mL) to obtain the supernatant, and then 1 M potassium iodide (KI) and potassium phosphate buffer (PPB: 1 mL) was added into the supernatant and allowed to sit for 30 min, and then the absorbance was noted at 390 nm. For the determination of MDA contents, plant leaf samples were homogenized by adding TCA (5 mL) and centrifuged at 12,000 rpm for 15 min. Afterward, the supernatant was added to 5 mL of thiobarbituric acid (TBA) and boiled at 100 °C for 30 min, then cooled quickly, and absorbance was measured at 532 nm to determine MDA concentration.

### 2.6. Determination of Antioxidant Activities

The method of Aebi [41] was used to determine the catalase (CAT) activity. We used 0.5 g of wheat leaf sample and blended it with 5 mL of PPB, then centrifuged the mixture for 15 min at 1000 rpm, and absorbance was measured at 240 nm to determine CAT activity. In the case of ascorbate peroxidase (APX), 0.5 g leaf sample was taken and homogenized in 5 mL potassium phosphate buffer (PPB: PH 7.8) using a mortar and pestle. Afterward, the prepared solution was centrifuged for 15 min at 10,000 rpm and absorbance was recorded at 290 nm to determine APX activity [42]. To determine peroxidase (POD) activity, 0.5 g leaf sample was homogenized in PPB (5 mL) with mortar and pestle, after which solution was centrifuged for 15 min at 10,000 rpm and the absorbance was recorded at 470 nm to determine POD activity [43]. In the case of ascorbic acid activity (AsA), 0.5 g plant samples were homogenized in 5 mL of trichloro-acetic acid (TCA) and centrifuged for 10 min to obtain the supernatant, and then absorbance was noted to determine AsA activity [43]. Lastly, to determine SOD activity, a reaction mixture of 400 µL H_2_O_2_, 25 mL buffer, 100 µL Triton, 50 µL sample, and 50 µL riboflavin was prepared and absorbance was measured at 560 for the determination of SOD activity [44].

### 2.7. Ionic Concentration

The wheat plant samples oven-dried (65 °C) and ground into powder. Afterward, 0.5% of ground sample was digested at 180 °C in a mixture containing two acids (hydrochloric acid, HCl, and nitric acid, HNO_3_) at a 1:2 ratio. Then, samples were filtered and diluted by adding the water. The concentration of Na, Ca, Mg, and K was determined using a flame photometer, and the concentration of Cl was determined using a chloride analyzer.

### 2.8. Determination of Yield Traits

The spike-bearing tillers were counted on every plant and the average calculated. Similarly, spikes per plant were counted on each plant and an average was calculated, while 10 spikes from each pot were used to determine the spike length and grains per spike. Lastly, the plants in each pot were harvested and biological yield was measured, and later the plants were threshed to determine grain yield.

### 2.9. Data Analysis

The data obtained from the collected traits were analyzed using a two-way analysis of variance (ANOVA) for wheat cultivars, stress treatments, and their interactions, and the least significant difference (LSD) was used at a 5% probability level to determine significance among treatments [45]. The results were achieved using a sigma plot, and principal component analysis was performed using R-studio.

## 3. Results

### 3.1. Growth and Morphological Traits

The results indicate that different stress treatments negatively affected growth parameters; similarly, cultivars also behaved differently in terms of growth and morphological traits (Table 1). Taller plants (54.77 cm) with more leaves per plant (LPP: 11.75) were recorded after treatment with Si, and shorter plants with low LPP were observed in case of combined salinity and heat stress (Table 1). In terms of cultivars, taller plants with more leaves were recorded in Akbar-2019, while shorter plants with the lowest number of leaves were recorded in Faisalabad-2008 (Table 1). Regarding interactions, the tallest plants with the most leaves were recorded in Akbar-2019 after Si application, and shorter plants with fewer leaves were recorded in Faisalabad-2008 with the combined imposition of heat and salinity stress (Table 1). The highest root fresh weight (RFW), shoot fresh weight (SFW), root dry weight (RDW), and shoot dry weight (SDW) were recorded in plants treated with Si without any stress, and the lowest values for these parameters were recorded in plants growing under combined heat and salinity stress (Table 1). Regarding interactions, the cultivar Akbar-2019 performed well with the highest RFW, SFW, RDW, and SDW parameters, and Faisalabad-2008 performed poorly, with the lowest RFW, SFW, RDW, and SDW parameters (Table 1).

### 3.2. Photosynthetic Pigments, Relative Water Contents, and Electrolyte Leakage

**Figure 1:**
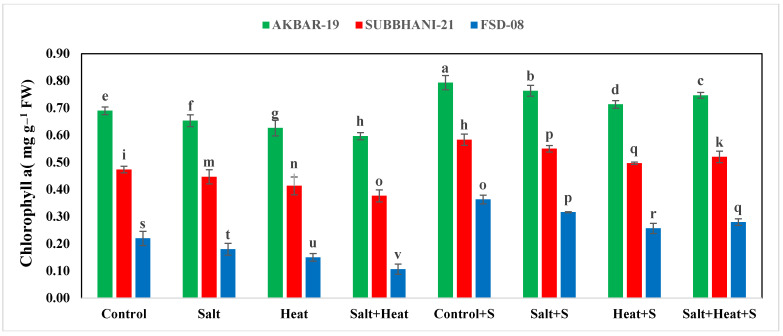

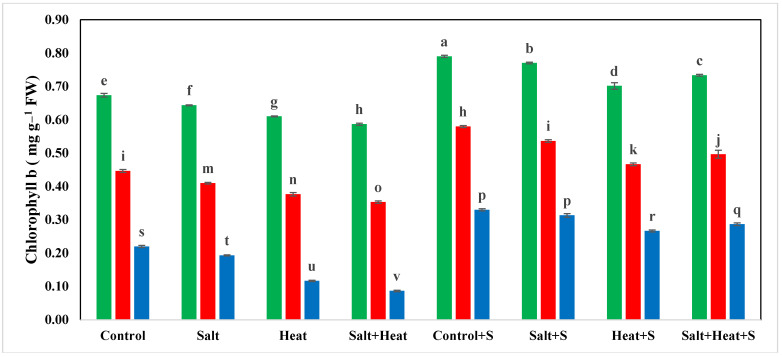
Effect of Si application on chlorophyll−a and chlorophyll−b contents of different wheat cultivars grown under salinity and heat stress conditions. The values given the figures are the means of three replicates with ± SD, and different letters indicate significance at *p* < 0.05.

The results indicate that heat and saline conditions significantly reduced the photosynthetic pigments and RWC of wheat plants (Table 1). The maximum chlorophyll-a (0.79 mg g^−1^ FW), chlorophyll-b (0.79 mg g^−1^ FW), and carotenoid (4.83 mg g^−1^ FW) values were recorded after Si application without any stresses, and the lowest values for chlorophyll-a (0.10 mg g^−1^ FW), chlorophyll-b (0.08 mg g^−1^ FW), and carotenoid (1.23 mg g^−1^ FW) were recorded in combined heat and salinity stress conditions without Si application. In terms of cultivars, the highest chlorophyll and carotenoid concentrations were identified in Akbar-2019 and the lowest was observed in Faisalabad-2008. The lowest chlorophyll-a content was recorded in Faisalabad-2008 (Figure 1).

Regarding the interactive effects, the highest values for photosynthetic pigments were recorded in Akbar-2019 after Si application, and the lowest were observed in Faisalabad-2008 under combined heat and salinity stress (Table 2). The variable stress treatments and cultivars also had a significant impact on RWC and EL. The highest RWC was recorded in Akbar-2019 after silicon application and the minimum was recorded in Faisalabad-2008 under combined heat and salinity stress conditions (Table 2). Conversely, maximum EL was recorded in Faisalabad-2008 under combined heat and salinity stresses, and the lowest EL was recorded for Akbar-2019 after the application of Si (Table 2).

### 3.3. Osmo-Protectants, Antioxidant Activities, and Oxidative Stress Causes

**Figure 2:**
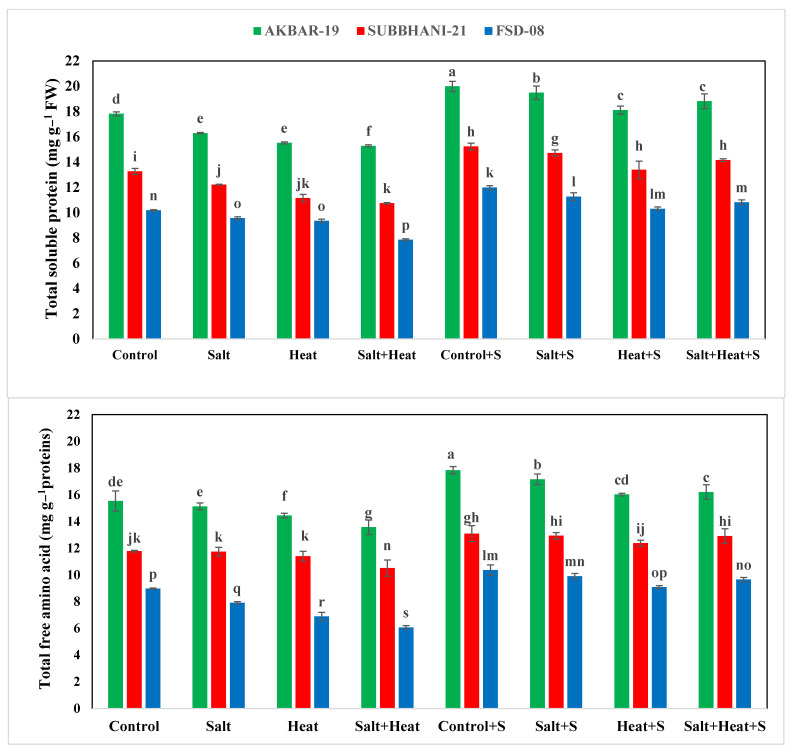
Effect of Si application on total soluble protein and free amino acids of different wheat cultivars grown under salinity and heat stress conditions. The values given in the figures are the means of three replicates with ± SD, and different letters indicate significance at *p* < 0.05.

**Figure 3:**
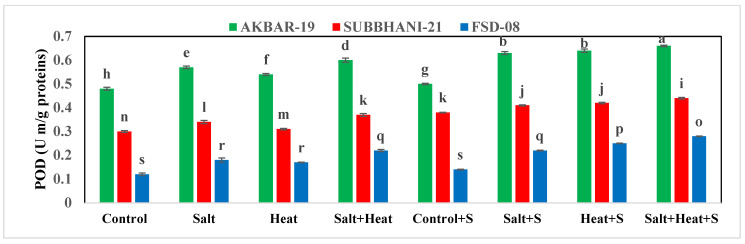

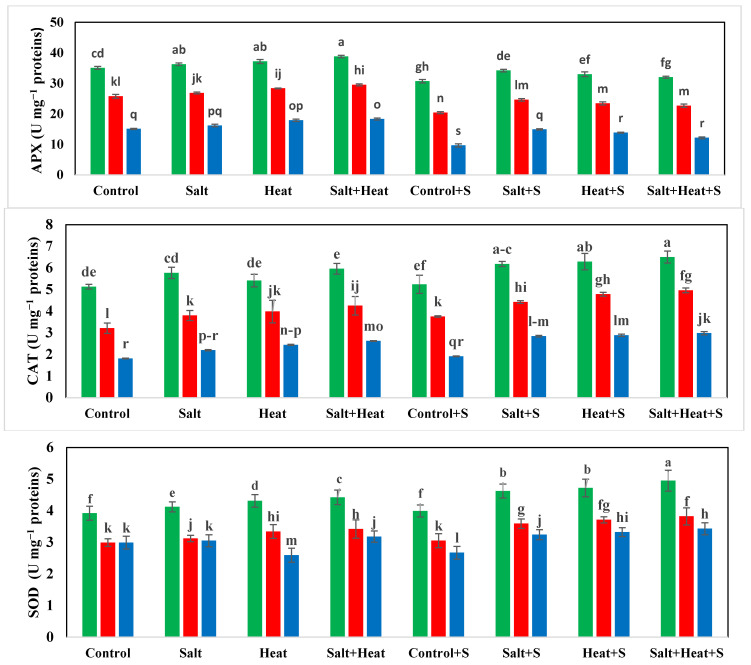
Effect of Si application on antioxidant activities of different wheat cultivars grown under salinity and heat stress conditions. APX: ascorbate peroxidase; CAT: catalase; POD: peroxidase; SOD: superoxide dismutase. The values given in the figures are the means of three replicates with ± SD, and different letters indicate significance at *p* < 0.05.

The highest TSP (15.73 mg g^−1^ protein) and FAA (19.99 mg g^−1^ protein) were recorded with Si treatment, and the lowest TSP and FAA were recorded when plants were subjected to heat and saline conditions (Figure 2). For the interaction effect, the highest TSP and FAA were observed in Akbar-2019 after Si supplementation, and the lowest TSP and FAA were recorded in Faisalabad-2008 under saline and heat conditions without the use of Si Figure 2). These results indicate that different treatments had significant impacts on all tested antioxidant activities. The activities of APX, POD, CAT, and SOD were significantly increased under stress conditions, and the highest activities of all these antioxidants (APX, POD, CAT, and SOD) were recorded under heat and saline conditions. After the application of Si under control conditions, the lowest antioxidant activities (APX, POD, CAT, and SOD) were recorded (Figure 3).

Similarly, oxidative stress markers (MDA and H_2_O_2_) also revealed a substantial increase in saline, heat stress, and combined saline and heat stress conditions; however, Si offset MDA and H_2_O_2_ accumulation by increasing antioxidant activities. In terms of cultivars, Akbar-2019 displayed the highest APX, POD, CAT, and SOD activities, which resulted in a substantial decrease in MDA and H_2_O_2_ production, while Faisalabad-2008 displayed the highest MDA and H_2_O_2_ accumulation (Table 3).

### 3.4. Yield Traits

The highest number of tillers per plant (TPP: 13.76), grains per spike (GPS: 58.91), and spike lengths (SL: 9.84 cm) were recorded after Si application under normal conditions and the lowest number of TPP, GPS, and SL were recorded in conditions where salt and heat stress were applied to plants without Si application (Table 4). In terms of cultivars, the highest TPP, GPS, and SL were recorded in Akbar-2019, followed by Subhani-2021, and the lowest TPP, GPS, and SL were recorded in Faisalabad-2008 (Table 4). Regarding interactions, Akbar-2019 again exhibited the highest TPP, GPS, and SL after Si application, and Faisalabad-2008 displayed the lowest TPP, GPS, and SL under combined heat and saline conditions without Si application (Table 4). The application of Si alone resulted in the highest 100-grain weight (100-GW: 8.82 g), grain yield per plant (GYPP: 9.79 g), and biological yield per plant (BYPP: 29.50 g) without any stresses, and the lowest 100-GW (5.45 g), GYPP (5.33 g), and BYPP (17.60 g) were recorded under conditions of combined heat and salinity stress (Table 4). In terms of cultivars, Akbar-2019 displayed the highest 100-GW, GYPP, and BYPP, and Faisalabad-2008 exhibited the lowest 100-GW, GYPP, and BYPP (Table 4).

### 3.5. Nutrient Concentration

**Figure 4:**
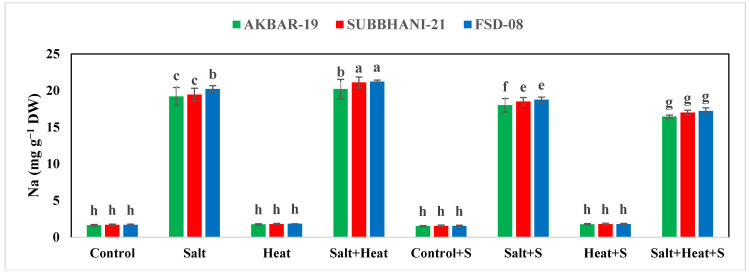

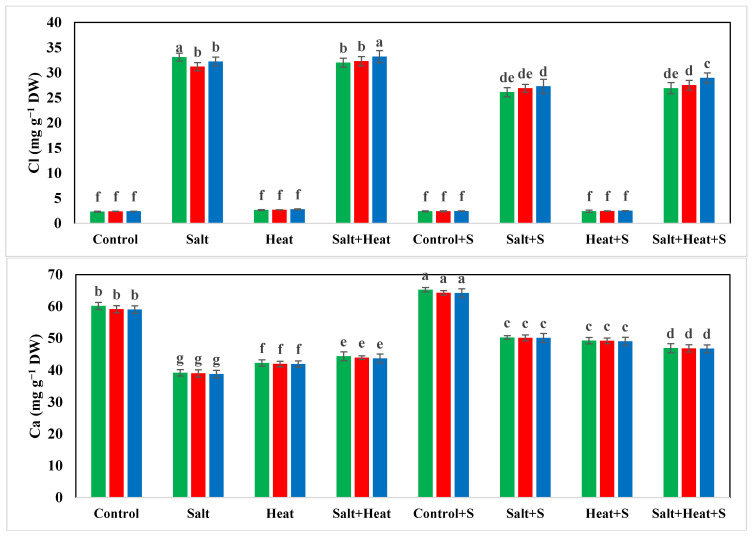
Effect of Si application on the Na, Cl, and Ca concentrations of different wheat cultivars grown under salinity and heat stress conditions. Na: sodium; Cl: chloride; Ca: calcium; DW: dry weight. The values given in the figures are the means of three replicates with ± SD, and different letters indicate significance at *p* < 0.05.

**Figure 5:**
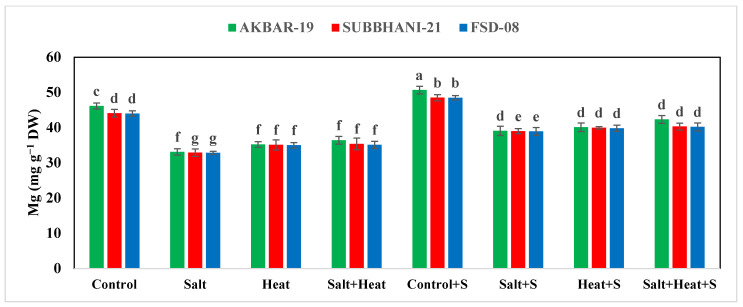

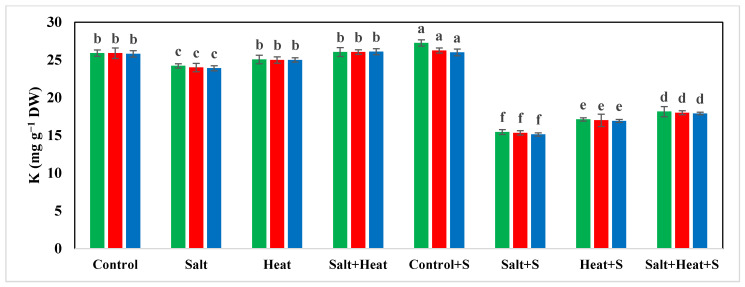
Effect of Si application on Mg and K concentrations of different wheat cultivars grown under salinity and heat stress conditions. Mg: magnesium; K: potassium; DW: dry weight. The values given in the figures are the means of three replicates with ± SD, and different letters indicate significance at *p* < 0.05.

The results indicate a substantial reduction in the concentration of Ca, Mg, and K, while an increase in the concentration of Na^+^ and Cl^−^ under salinity and heat stress conditions (Figure 4). The highest Ca, Mg, and K concentrations were recorded in plants grown under normal conditions with the application of Si, and the lowest Ca, Mg, and K concentrations were recorded in plants grown under saline and heat stress conditions (Figure 4 and Figure 5). The application of Si offset the negative effects of saline and heat stress and considerably increased the uptake of Ca, Mg, and K under both saline and heat conditions (Table 5). The cultivar type had a non-significant impact on Ca, Mg, and K concentration; however, Akbar-2019 displayed slightly higher nutrient concentrations in plant parts (Figure 4 and Figure 5). The highest Na^+^ and Cl^−^ concentrations were recorded under combined saline and heat stress conditions and were comparable with saline stress alone, and the lowest Na^+^ and Cl^−^ concentrations were recorded in control conditions with Si application (Figure 4 and Figure 5).

### 3.6. Principle Component Analysis

**Figure 6:**
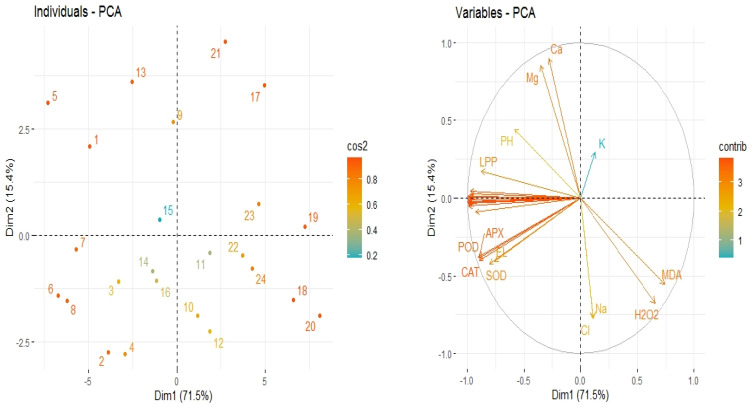
The scores on left and loading plots on right of the principal component analysis (PCA) show the effect of diverse treatments on the studied traits.

The collected data set on different traits were subjected to principal component analysis (PCA) in order to determine the relationships of the studied traits. The analysis given in Figure 6 indicates that two components (PC_1_ and PC_2_) showed 86.9% total variance, where PC_1_ comprised 71.5%, and PC_2_ comprised 15.4% (Figure 6). PCA findings indicated that salinity and heat stress induced negative impacts on the growth and yield traits; however, Si application mitigated the adverse effects of both these stresses. There was a negative relationship between stress treatments and growth and yield traits; RWC; chlorophyll concentration; and Mg, Ca, and K uptake and there was positive association between the stress treatments and antioxidant activities; MDA; H_2_O_2_; EL; and Na^+^ and Cl^−^ accumulation (Figure 6).

## 4. Discussion

Heat and salinity stresses are serious co-occurring abiotic stresses that negatively affect plant growth and development. Thus, it is essential to understand the response of plants against both these abiotic stresses [46,47]. In the current study, SS significantly reduced the growth and biomass of all the cultivars, but the most adverse impacts of SS were reported for Faisalabad-2008. This indicates that salinity adversely affects the growth and biomass production in the salt-sensitive cultivar Faisalabad-2008, as compared to salt-tolerant cultivars, and it has been well documented that SS causes a significant reduction in the growth of salt-sensitive cultivars [48,49]. The salinity-induced reduction in wheat cultivars was linked to Na^+^-induced ionic toxicity [50] and increased MDA and H_2_O_2_ production (Table 1). The salt stress in the growing medium significantly increased Na^+^ accumulation; however, salt-tolerant cultivars accumulated less Na^+^, which might be an important reason for the marked improvement in growth and biomass of this cultivar.

Salinity toxicity also reduced photosynthetic pigments and RWC and increased MDA and H_2_O_2_ accumulation. Higher concentrations of salts induced oxidative stress, which damaged chlorophyll enzymes and increased the activity of chlorophyll-degrading enzymes, thus resulting in less chlorophyll synthesis [50]. Moreover, SS also reduced RWC due to its detrimental effects on water absorption and water availability from soil, which affects the overall water status of plants [50]. Likewise, SS also increased the EL of leaves due to the overproduction of ROS, which disrupts membrane integrity and results in an increase in EL under high-saline conditions [28]. The results indicate that antioxidant activities were increased in high-saline conditions, and Akbar-2019 displayed the most antioxidant activities, while Faisalabad-2008 exhibited fewer antioxidant activities. These results are same as the outcomes of different authors, who also found a marked increase in the antioxidant activities of salt-tolerant cultivars, as compared to salt-sensitive cultivars [51,52,53,54,55].

HS hampered the growth of wheat cultivars; nonetheless, the severity of HS depends on the intensity of HS and the genotypes [56,57,58]. In this study, HS induced more negative effects on the cultivar Faisalabad-2008 as compared to Akbar-2019. HS reduced the chlorophyll synthesis of wheat cultivars, possibly due to excessive MDA and H_2_O_2_ production and the increase in the activity of chlorophyll-degrading enzyme activities [59,60]. The generation of ROS is considered to play a key role in the activation of the mechanism leading to a plant’s adaptation to stress conditions [61], and the activation of antioxidants is considered an important response to scavenging ROS. However, other factors, such as osmolyte accumulation, play an important role in heat tolerance. In the present study, the accumulation of TSP and FAA increased under HS, and the cultivar Akbar-2019 showed the highest TSP and FAA concentrations. The increased concentrations of TSP and FAA maintained a cell redox balance by increasing antioxidant activities, which ensured better chlorophyll synthesis and the production of assimilates and resulted in a significant increase in plant growth [28]. Moreover, HS also caused a marked reduction in yield traits; however, less reduction was found in Akbar-2019. The cultivar Akbar-2019 was heat tolerant, and it displayed better antioxidant activities, osmolyte accumulation, and nutrient uptake (Ca, Mg, and K), which resulted in better yields and yield traits in this cultivar. Our results are same as the findings of different authors, who also found that salt-tolerant cultivars displayed a better performance when compared to salt-sensitive cultivars [62,63].

Salinity induces a marked reduction in the growth of wheat cultivars via the excessive build-up of Na^+^ and Cl^−^ and reducing nutrient uptake and photosynthetic performance [64,65,66]. However, the application of Si significantly improved wheat growth and yield under saline conditions. The exogenous application of Si increases the endogenous Si level, which protect the plants from the damages of salt stress by reducing Na^+^ uptake, ROS production, and EL and increasing water and nutrient uptake, thus ensuring better plant growth [29,67,68]. Si substantially increased antioxidant activity and osmolyte accumulation (TSP and FAA), which neutralize the effects of oxidative stress in plants by protecting them from cell outbursts, thus resulting in better plant performance [28,67]. The increase in antioxidant activity and osmolyte accumulation indicates that Si-induced cellular signaling boosts the plant’s endogenous defense system to counter the toxic effects of SS. The possible mechanism of the Si-induced increase in antioxidant activities is due to increased K^+^ uptake, reduced Na^+^ uptake, and increased water and nutrient uptake [69,70,71,72]. However, a knowledge gap still exists in terms of clarifying the interaction of exogenously applied Si and the antioxidants in wheat plants growing under SS conditions.

The results indicate that HS caused a marked reduction in wheat growth; however, exogenous Si mitigated the adverse impacts of HS. The application of Si increases chlorophyll synthesis, maintains plant water stats, reduces ROS production and EL, triggers antioxidant activities, and stimulates osmolyte accumulation, which counters the toxic effects of HS and ensures better plant growth under stress conditions [73,74,75]. In this study, the activity of all antioxidants and osmolytes was significantly increased in wheat cultivars under HS. This indicates that Si triggers cell signaling, which activates the antioxidant defense system and increases the accumulation of potential osmolytes to counter the toxic effects of HS, which has been well documented in the literature by various authors [76,77,78].

We also found that combined heat and SS markedly reduced the growth of wheat cultivars. The combined imposition of heat and saline conditions markedly reduced chlorophyll contents by increasing H_2_O_2_ production and enzyme activity involved in chlorophyll degradation [79,80,81,82,83]. However, Si application enabled the better chlorophyll and carotenoid synthesis due to less ROS production, more antioxidant activities, better stay-green traits, and higher leaf area [80,81]. The results indicate that saline and heat conditions increase the TSP and FAA of wheat cultivars. Furthermore, Si application increased TSP and FAA, which could be ascribed to increased protein kinase synthesis and improved cellular signaling, which stimulated TSP and FAA accumulation to counter the effects of heat and SS. Si application is considered to be very useful in normalizing osmolytes synthesis, which ensures proper plant functioning and better growth under stress conditions [84,85]. Higher antioxidant activities are important means for a plant to tolerate abiotic stresses. In the present study, Si application significantly increased all antioxidants in wheat cultivars. These results are the same as the findings of many studies where authors found that Si application increased antioxidant activities and neutralized oxidative stress in plants under high heat and saline conditions [86]. However, the mechanisms by which Si increases antioxidants under combined heat and saline conditions are still unknown. Therefore, in-depth studies are needed to underpin how Si affects the mechanism of the antioxidant system to induce combined heat and salinity tolerance in plants.

We noted that the accumulation of Na^+^ and Cl^−^ was significantly increased under combined heat and saline conditions (Figure 5 and 6), while the uptake of K^+^ was significantly decreased under HS and SS, which could be due to an increase in K^+^ efflux [87]. However, this mechanism remains to be confirmed, although this efflux of K^+^ can be reduced via the application of osmo-protectants and nutrients application [88]. In the present study, the application Si improved the K^+^ uptake under combined heat and saline conditions, which could be due to a substantial decrease in the K^+^ efflux. The results also indicate that saline and heat conditions cause a significant reduction in Ca, Mg, and K uptake and application, and Si significantly increases the uptake of the aforementioned nutrients (Table 5). However, it is still necessary to explore the mechanism through which Si increases the uptake and accumulation of these aforementioned nutrients in wheat under combined heat and saline conditions. The findings of the present study indicate that combined heat and salinity stress conditions significantly decreased the growth and yield of wheat plants (Table 1 and Table 5). Nonetheless, Si application significantly increased the yield of wheat under combined heat and saline conditions, which can be ascribed to improved plant physiological functioning, leaf water status, antioxidant activities, metabolic activities, nutrient absorption, osmolyte accumulation, and the restricted uptake of Na^+^ and Cl^−^ [89].

## 5. Conclusions

In conclusion, combined heat and saline conditions significantly reduce wheat growth and final yield by decreasing leaf water status, photosynthetic pigments, osmolyte accumulation, and nutrient uptake. Nonetheless, exogenous Si supplementation mitigated the deleterious impacts of both saline and heat stress and improves the yield of wheat by increasing nutrient uptake, leaf water status, photosynthetic pigments, osmolyte accumulation, and antioxidant activities. Thus, the application of Si could be an effective approach for minimizing the toxic effects of combined heat and salinity stress in wheat. However, more field studies under a wide range of climate conditions are urgently needed to optimize the rate of exogenous Si application for wheat crops. In addition, more metabolomic, proteomic, and transcriptomic studies are required to discover the mechanism behind the mitigation of heat and salinity stress in wheat via silicon application. Additionally, in-depth studies are also needed to explore how silicon effects the mechanism of antioxidant systems and ion absorption to induce heat and salinity-tolerance in plants.

## Figures and Tables

**Table 1:** Effect of Si application on growth traits of different wheat cultivars grown under salinity and heat stress conditions.

Wheat Cultivars	Treatments	PH (cm)	LPP	RFW (g)	SFW (g)	RDW (g)	SDW (g)
Akbar-2019	Control	53.00 ± 0.47 c	10.93 ± 0.32 df	9.90 ± 0.12 d	14.30 ± 0.19 d	8.43 ± 0.09 c	6.58 ± 0.037 d
	Salt stress (8 dS m^−1^)	43.66 ± 0.55 gh	10.50 ± 0.29 ef	9.46 ± 0.21 c	14.26 ± 0.21 d	8.26 ± 0.12 d	6.44 ± 0.019 f
	Heat stress	41.33 ± 0.29 hi	9.23 ± 0.20 g	8.83 ± 0.15 f	13.23 ± 0.13 e	7.93 ± 0.021 e	6.35 ± 0.020 f
	Salt + Heat	38.66 ± 0.48 ij	8.50 ± 0.22 hi	8.26 ± 0.20 g	13.13 ± 0.21 e	7.81 ± 0.14 f	6.21 ± 0.022 g
	Si (500 mg kg^−1^)	58.00 ± 0.72 a	14.50 ± 0.12 a	12.93 ± 0.7 a	16.40 ± 0.25 a	8.84 ± 0.10 a	6.95 ± 0.015 a
	Salt + Si	57.00 ± 0.63 ab	14.16 ± 0.29 ab	11.36 ± 0.12 b	15.43 ± 0.15 b	8.75 ± 0.08 a	6.84 ± 0.029 b
	Heat + Si	53.33 ± 0.55 c	11.50 ± 0.28 cd	10.83 ± 0.14 c	14.43 ± 0.19 cd	8.56 b ± 0.10 b	6.62 ± 0.031 d
	Salt + Heat + Si	54.66 b ± 0.49 bc	13.83 ± 0.42 b	11.00 ± 0.19 bc	14.80 ± 0.32 c	8.64 ± 0.12 b	6.71 ± 0.03 c
Subhani-2021	Control	45.33 ± 0.33 fg	10.23 ± 0.12 f	7.66 ± 0.20 ij	10.86 ± 0.12 hi	7.34 ± 0.024 i	5.62 ± 0.049 k
	Salt stress (8 dS m^−1^)	45.00 ± 0.66 fg	8.60 ± 0.22 hi	7.30 ± 0.22 k	10.43 ± 0.09 ij	6.80 ± 0.10 j	5.38 ± 0.018 l
	Heat stress	40.00 ± 0.82 ij	8.16 ± 0.24 i-k	6.60 ± 0.18 l	10.06 ± 0.18 gh	6.78 ± 0.09 j	5.16 ± 0.026 m
	Salt + Heat	37.00 ± 0.33 j	7.10 ± 0.29 m	6.26 ± 0.15 m	9.70 ± 0.06 jk	6.64 ± 0.014 k	6.96 ± 0.012 n
	Si (500 mg kg^−1^)	54.66 ± 0.22 bc	11.93 ± 0.32 c	8.80 ± 0.09 gh	12.93 ± 0.32 e	7.82 ± 0.019 f	6.12 ± 0.040 h
	Salt + Si	53.33 ± 0.19 c	10.66 ± 0.13 ef	7.40 ± 0.12 jk	12.06 ± 0.38 f	7.62 ± 0.020 g	5.87 ± 0.020 i
	Heat + Si	51.66 ± 0.38 cd	10.26 ± 0.42 f	7.33 ± 0.05 k	11.06 ± 0.12 gh	7.46 ± 0.12 h	5.64 ± 0.019 k
	Salt + Heat + Si	52.66 ± 0.42 c	10.63 ± 0.21 ef	7.93 ± 0.12 hi	11.46 ± 0.14 g	7.53 ± 0.10 gh	5.73 ± 0.064 j
Faisalabad-2008	Control	47.00 ± 0.26 ef	7.60 ± 0.34 k-m	4.96 ± 0.22 p	6.73 ± 0.06 o	5.77 ± 0.05 op	4.28 ± 0.042 r
	Salt stress (8 dS m^−1^)	46.00 ± 0.48 fg	7.33 ± 0.42 lm	4.76 ± 0.24 pq	5.96 ± 0.10 p	5.69 ± 0.07 pq	3.91 ± 0.032 s
	Heat stress	41.66 ± 0.55 hi	7.23 ± 0.37 lm	4.53 ± 0.15 q	4.83 ± 0.05 q	5.64 ± 0.08 q	3.61 ± 0.074 t
	Salt + Heat	31.00 ± 0.41 k	7.20 ± 0.22 m	3.86 ± 0.11 r	3.56 ± 0.06 r	5.38 ± 0.012 r	3.43 ± 0.09 u
	Si (500 mg kg^−1^)	51.66 ± 0.56 cd	8.83 ± 0.19 gh	6.13 ± 0.07 m	9.16 ± 0.14 l	6.45 ± 0.029 l	4.77 ± 0.11 o
	Salt + Si	49.33 ± 0.43 df	8.30 ± 0.28 h-j	5.80 ± 0.08 n	8.56 ± 0.12 m	6.30 ± 0.026 m	4.61 ± 0.025 p
	Heat + Si	47.33 ± 0.40 ef	7.80 ± 0.22 j-l	5.46 ± 0.04 o	7.90 ± 0.10 n	5.84 ± 0.041 o	4.33 ± 0.013 r
	Salt + Heat + Si	47.66 ± 0.59 ef	8.03 ± 0.20 i-k	5.70 ± 0.11 no	8.20 ± 0.16 mn	6.12 ± 0.033 n	4.43 ± 0.021 p

PH: plant height; LPP: leaves per plant; RFW: root fresh weight; SFW: shoot fresh weight; RDW: root dry weight; SDW: shoot dry weight. The values given in the table are the mean of three replicates with ± SD (standard deviation), and different letters indicate significance at *p* < 0.05.

**Table 2:** Effect of Si application on photosynthetic pigments and physiological traits of different wheat cultivars grown under salinity and heat stress conditions.

Wheat Cultivars	Treatments	Carotenoids (mg g^−1^ FW)	Electrolyte Leakage (%)	Relative Water Contents (%)
Akbar-2019	Control	4.34 ± 0.019 c	54.45 ± 1.26 c	79.94 ± 0.34 d
	Salt stress (8 dS m^−1^)	4.18 ± 0.035 cd	56.11 ± 1.15 b	78.78 ± 1.16 df
	Heat stress	4.08 ± 0.060 df	57.17 ± 1.29 b	77.89 ± 1.38 e–g
	Salt + Heat	3.99 ± 0.087 ef	58.58 ± 0.78 a	77.14 ± 0.89 fg
	Si (500 mg kg^−1^)	4.83 ± 0.015 a	50.39 ± 0.98 f	86.48 ± 0.38 a
	Salt + Si	4.60 ± 0.021 b	54.27 ± 0.56 cd	84.26 ± 0.37 b
	Heat + Si	4.36 ± 0.012 c	53.10 ± 1.10 de	82.36 ± 0.46 c
	Salt + Heat + Si	4.69 ± 0.020 ab	51.90 ± 0.78 e	84.54 ± 0.66 b
Subhani-2021	Control	3.26 ± 0.016 h	47.33 ± 0.97 hi	70.16 ± 0.56 j
	Salt stress (8 dS m^−1^)	3.15 ± 0.11 h	48.10 ± 0.42 gh	68.89 ± 0.78 j
	Heat stress	2.82 ± 0.062 i	48.97 ± 1.10 g	65.42 ± 0.89 k
	Salt + Heat	2.68 ± 0.014 ij	50.40 ± 0.92 f	63.37 ± 0.33 l
	Si (500 mg kg^−1^)	3.83 ± 0.029 f	42.57 ± 0.42 l	78.59 ± 0.35 df
	Salt + Si	3.59 ± 0.022 g	46.24 ± 0.31 ij	76.48 ± 1.11 g
	Heat + Si	3.25 ± 0.040 h	45.42 ± 0.98 j	72.3 ± 0.88 i
	Salt + Heat + Si	3.48 ± 0.034 g	43.99 ± 1.14 k	74.39 ± 0.78 h
Faisalabad-2008	Control	1.90 ± 0.022 n	37.98 ± 1.29 no	49.43 ± 0.36 p
	Salt stress (8 dS m^−1^)	1.55 ± 0.031 o	39.04 ± 1.41 n	46.17 ± 0.99 q
	Heat stress	1.35 ± 0.025 p	40.90 ± 1.56 m	44.94 ± 0.89 q
	Salt + Heat	1.23 ± 0.02 p	42.72 ± 1.12 l	41.36 ± 1.12 r
	Si (500 mg kg^−1^)	2.56 ± 0.022 jk	34.30 ± 1.14 q	59.48 ± 0.88 m
	Salt + Si	2.40 ± 0.016 kl	36.92 ± 1.38 op	57.99 ± 0.99 m
	Heat + Si	2.16 ± 0.011 m	36.29 ± 1.42 p	52.46 ± 0.99 o
	Salt + Heat + Si	2.25 ± 0.012 lm	35.33 ± 1.56 pq	54.72 ± 1.12 n

The values given in the table are the means of three replicates with ± SD, and different letters indicate significance at *p* < 0.05.

**Table 3:** Effect of Si application on oxidative stress markers of different wheat cultivars grown under salinity and heat stress conditions.

Wheat Cultivars	Treatments	MDA (µ mol g^−1^ FW)	H_2_O_2_ µ mol g^−1^ FW)
Akbar-2019	Control	3.92 ± 0.12 n	2.62 ± 0.26 j
	Salt stress (8 dS m^−1^)	4.81 ± 0.41 ij	3.49 ± 0.21 f
	Heat stress	4.92 ± 0.10 j	3.61 ± 0.09 ef
	Salt + Heat	5.11 ± 0.22 i	3.72 ± 0.10 e
	Si (500 mg kg^−1^)	3.70 ± 0.31 p	2.50 ± 0.19 k
	Salt+ Si	4.52 ± 0.15 l	3.39 ± 0.10 g
	Heat + Si	4.60 ± 0.30 k	3.41 ± 0.12 g
	Salt + Heat + Si	4.72 ± 0.21 jk	3.52 ± 0.29 f
Subhani-2021	Control	4.02 ± 0.10 o	2.83 ± 0.22 i
	Salt stress (8 dS m^−1^)	5.59 ± 0.14 g	4.13 ± 0.10 v
	Heat stress	5.70 ± 0.11 g	4.19 ± 0.15 v
	Salt + Heat	5.88 ± 0.40 f	4.32 ± 0.12 b
	Si (500 mg kg^−1^)	3.81 ± 0.19 p	2.72 ± 0.21 i
	Salt + Si	5.20 ± 0.12 i	3.86 ± 0.18 ef
	Heat + Si	5.34 ± 0.33 h	3.92 ± 0.15 e
	Salt + Heat + Si	5.42 ± 0.22 h	4.04 ± 0.20 d
Faisalabad-2008	Control	4.22 ± 0.12 m	2.99 ± 0.31 h
	Salt stress (8 dS m^−1^)	6.92 ± 0.19 b	4.62 ± 0.19 a
	Heat stress	7.02 ± 0.31 b	4.70 ± 0.22 a
	Salt + Heat	7.14 ± 0.22 a	4.72 ± 0.15 a
	Si (500 mg kg^−1^)	3.98 ± 0.18 n	2.80 ± 0.11 i
	Salt + Si	6.51 ± 0.26 e	4.33 ± 0.20 b
	Heat + Si	6.65 ± 0.14 d	4.41 ± 0.14 b
	Salt + Heat + Si	6.80 ± 0.10 c	4.52 ± 0.19 ab

MDA: malondialdehyde; H_2_O_2_: hydrogen peroxide. The values given in the table are the mean of three replicates with ± SD, and different letters indicate significance at *p* < 0.05.

**Table 4:** Effect of Si application on the yield traits of different wheat cultivars grown under salinity and heat stress conditions.

Wheat Cultivars	Treatments	TPP	GPS	SL (cm)	100 GW (g)	GYPP (g)	BYPP (g)
Akbar-2019	Control	11.63 ± 0.04 d	50.97 ± 0.69 e	9.57 ± 0.018 d	8.22 ± 0.035 d	9.17 ± 0.10 c	27.60 ± 0.23 e
	Salt stress (8 dS m^−1^)	11.16 ± 0.03 e	50.20 ± 0.21 ef	9.51 ± 0.012 df	8.15 ± 0.023 d	8.81 ± 0.23 d	27.41 ± 0.10 f
	Heat stress	10.26 ± 0.09 f	50.09 ± 0.54 ef	9.44 ± 0.024 e	7.95 ± 0.062 e	8.56 ± 0.14 e	26.63 ± 0.11 g
	Salt + Heat	10.06 ± 0.02 fg	49.88 ± 0.29 f	9.36 ± 0.046 f	7.77 ± 0.090 f	8.31 ± 0.22 f	26.35 ± 0.11 h
	Si (500 mg kg^−1^)	13.76 ± 0.10 a	58.91 ± 0.34 a	9.84 ± 0.052 a	8.82 ± 0.054 a	9.79 ± 0.15 a	29.50 ± 0.17 a
	Salt + Si	13.23 ± 0.09 b	56.53 ± 0.41 b	9.81 ± 0.11 ab	8.66 ± 0.042 b	9.69 ± 0.075 a	29.35 ± 0.22 b
	Heat + Si	11.83 ± 0.12 d	52.68 ± 0.65 d	9.67 ± 0.098 c	8.47 ± 0.061 c	9.29 ± 0.038 bc	28.42 ± 0.23 d
	Salt + Heat + Si	12.90 ± 0.14 c	47.83 ± 0.56 g	9.75 ± 0.012 b	8.50 ± 0.050 c	9.45 b ± 0.18 b	28.67 ± 0.17 e
Subhani-2021	Control	8.93 ± 0.10 j	39.55 ± 0.49 j	8.42 ± 0.033 jk	6.89 ± 0.038 j	7.29 ± 0.042 i	22.61 ± 0.056 m
	Salt stress (8 dS m^−1^)	8.46 ± 0.09 k	39.21 ± 0.62 jk	8.38 ± 0.11 k	6.72 ± 0.027 k	6.89 ± 0.015 j	21.50 ± 0.22 n
	Heat stress	8.26 ± 0.19 k	38.96 ± 0.54 jk	8.30 ± 0.012 l	6.46 ± 0.035 l	6.43 ± 0.12 k	21.22 ± 0.21 o
	Salt + Heat	7.80 ± 0.18 l	38.23 ± 0.48 k	8.14 ± 0.029 m	6.22 ± 0.10 m	6.21 ± 0.044 l	20.71 ± 0.18 p
	Si (500 mg kg^−1^)	9.83 ± 0.20 g	47.83 ± 0.50 g	9.22 ± 0.034 g	7.62 ± 0.078 g	7.90 ± 0.015 g	25.53 ± 0.32 i
	Salt+ Si	9.43 ± 0.14 h	46.43 ± 0.26 h	8.84 ± 0.012 h	7.35 ± 0.053 h	7.54 ± 0.044 h	24.66 ± 0.098 j
	Heat + Si	9.13 ± 0.010 ij	39.94 ± 0.39 j	8.46 ± 0.10 j	7.09 ± 0.042 i	7.42 ± 0.078 hi	23.57 ± 0.11 l
	Salt + Heat+ Si	9.36 ± 0.012 hi	44.90 ± 0.42 j	8.54 ± 0.018 i	7.13 ± 0.054 i	7.49 ± 0.065 h	24.35 ± 0.12 k
Faisalabad-2008	Control	5.26 ± 0.014 p	24.22 ± 0.31 p	7.20 ± 0.019 r	5.24 ± 0.029 q	4.89 ± 0.053 p	16.59 ± 0.13 u
	Salt stress (8 dS m^−1^)	4.80 ± 0.024 q	21.77 ± 0.46 q	7.14 ± 0.069 r	4.87 ± 0.49 r	4.42 ± 0.046 q	15.74 ± 0.29 v
	Heat stress	4.26 ± 0.039 r	19.73 ± 0.12 r	6.93 ± 0.12 s	4.52 ± 0.54 s	4.36 ± 0.035 q	14.59 ± 0.24 w
	Salt + Heat	3.83 ± 0.043 s	18.29 ± 0.19 s	6.74 ± 0.043 t	3.87 ± 0.042 t	3.93 ± 0.052 r	13.26 ± 0.12 x
	Si (500 mg kg^−1^)	6.66 ± 0.053 l	35.53 ± 0.29 l	7.94 ± 0.023 n	5.82 ± 0.031 m	5.87 ± 0.016 m	19.70 ± 0.13 q
	Salt + Si	6.23 ± 0.044 n	33.27 ± 0.25 m	7.82 ± 0.017 o	5.65 ± 0.064 n	5.62 ± 0.029 n	18.66 ± 0.22 r
	Heat + Si	5.06 ± 0.029 pq	29.83 ± 0.21 o	7.35 ± 0.015 q	5.38 ± 0.05 pq	5.26 ± 0.043 o	17.47 ± 0.24 t
	Salt + Heat + Si	5.66 ± 0.11 o	31.92 ± 0.14 n	7.49 ± 0.025 p	5.45 ± 0.039 p	5.33 ± 0.040 o	17.60 ± 0.36 s

TPP: total productive tillers; GPS: grains per spike; SL: spike length; GW: grain weight; GYPP: grain yield per plant; BYPP: biological yield per plant. The values given in the table are the means of three replicates with ± SD, and different letters indicate significance at *p* < 0.05.

**Table 5:** Effect of Si application on growth traits of different wheat cultivars grown under salinity and heat stress conditions cultivars.

Wheat Cultivars	Treatments	Na (mg g^−1^ DW)	Cl (mg g^−1^ DW)	Ca (mg g^−1^ DW)	Mg (mg g^−1^ DW)	K (mg g^−1^ DW)
Akbar-19	Control	1.64 ± 0.12 h	2.32 ± 0.12 f	60.12 ± 1.12 b	46.12 ± 0.85 c	25.90 ± 0.42 b
	Salt stress (8 dS m^−1^)	19.20 ± 1.22 c	33.10 ± 0.78 a	39.21 ± 1.00 g	33.10 ± 0.92 f	24.22 ± 0.29 c
	Heat stress	1.76 ± 0.10 h	2.65 ± 0.10 f	42.23 ± 0.99 f	35.22 ± 0.78 f	25.07 ± 0.56 b
	Salt + Heat	20.20 ± 1.34 b	32.00 ± 0.89 b	44.33 ± 1.42 e	36.41 ± 1.11 f	26.05 ± 0.60 b
	Si (500 mg kg^−1^)	1.52 ± 0.10 h	2.40 ± 0.10 f	65.23 ± 0.68 a	50.67 ± 1.09 a	27.25 ± 0.42 a
	Salt+ Si	18.00 ± 0.92 f	26.12 ± 0.92 f	50.24 ± 0.56 c	39.10 ± 1.32 d	15.45 ± 0.33 f
	Heat + Si	1.78 ± 0.10 h	2.42 ± 0.022 f	49.23 ± 0.99 c	40.11 ± 1.21 d	17.12 ± 0.22 e
	Salt+ Heat+ Si	16.44 ± 0.22 h	26.91 ± 1.11 de	46.88 ± 1.43 d	42.33 ± 1.11 d	18.15 ± 0.67 d
Subhani-21	Control	1.70 ± 0.49 h	2.38 ± 0.029 f	59.12 ± 1.14 b	44.10 ± 1.09 d	25.90 ± 0.70 b
	Salt stress (8 dS m^−1^)	19.44 ± 0.89 c	31.20 ± 0.82 b	39.00 ± 1.10 g	32.91 ± 1.05 g	24.00 ± 0.55 c
	Heat stress	1.80 ± 0.10 h	2.70 ± 0.002 f	41.91 ± 0.86 f	35.10 ± 1.22 f	25.00 ± 0.42 b
	Salt + Heat	21.10 ± 0.75 a	32.30 ± 0.89 b	43.90 ± 0.58 e	35.40 ± 1.10 f	26.05 ± 0.29 b
	Si (500 mg kg^−1^)	1.55 ± 0.40 h	2.41 ± 0.10 f	64.23 ± 0.72 a	48.50 ± 0.87 b	26.25 ± 0.33 a
	Salt+ Si	18.50 ± 0.56 e	26.88 ± 0.78 de	50.12 ± 0.92 c	38.99 ± 0.72 e	15.33 ± 0.29 f
	Heat + Si	1.80 ± 0.29 h	2.46 ± 0.022 f	49.20 ± 0.88 c	39.98 ± 0.33 d	17.00 ± 0.82 e
	Salt+ Heat+ Si	16.99 ± 0.33 g	27.50 ± 0.98 d	46.80 ± 1.13 d	40.30 ± 0.92 d	18.00 ± 0.26 d
Faisalabad-08	Control	1.70 ± 0.10 h	2.40 ± 0.020 f	59.00 ± 1.19 b	44.00 ± 0.76 d	25.80 ± 0.42 b
	Salt stress (8 dS m^−1^)	20.22 ± 0.45 b	32.20 ± 0.89 b	38.78 ± 1.21 g	32.85 ± 0.42 g	23.90 ± 0.33 c
	Heat stress	1.85 ± 0.013 h	2.79 ± 0.10 f	41.90 ± 0.99 f	35.00 ± 0.78 f	25.00 ± 0.29 b
	Salt + Heat	21.22 ± 0.22 a	33.20 ± 1.20 a	43.65 ± 1.40 e	35.11 ± 0.99 f	26.10 ± 0.40 b
	Si (500 mg kg^−1^)	1.55 ± 0.10 h	2.45 ± 0.033 f	64.20 ± 1.32 a	48.49 ± 0.72 b	26.00 ± 0.44 a
	Salt+ Si	18.76 ± 0.34 e	27.20 ± 1.41 d	50.10 ± 1.33 c	38.92 ± 0.88 e	15.12 ± 0.22 f
	Heat + Si	1.80 ± 0.10 h	2.50 ± 0.020 d	49.01 ± 1.29 c	39.83 ± 0.89 d	16.92 ± 0.20 e
	Salt+ Heat+ Si	17.22 ± 0.42 g	28.96 ± 0.99 c	46.72 ± 1.20 d	40.22 ± 1.10 d	17.90 ± 0.17 d

Na: sodium, Cl: chloride, Ca: calcium, Mg: magnesium, K: potassium, DW: dry weight. The values give in tables are mean of three replicates with ± and different letters indicating significant at *p* < 0.05.

## Data Availability

Not applicable.
